# A comparative study on the effectiveness of online and in-class team-based learning on student performance and perceptions in virtual simulation experiments

**DOI:** 10.1186/s12909-024-05080-3

**Published:** 2024-02-12

**Authors:** Jing Shen, Hongyan Qi, Ruhuan Mei, Cencen Sun

**Affiliations:** 1 Department of Pathology and Pathophysiology, Zhejiang University School of Medicine, 310058 Hangzhou, China; 2 Experimental Teaching Center of Basic Medicine, Zhejiang University School of Medicine, 310058 Hangzhou, China

**Keywords:** Team-based learning, Online, Face-to-face, Flipped classroom, Virtual simulation experiments, Medical education

## Abstract

**Background:**

The swift transition to online teaching in medical education has presented the challenge of replicating in-class engagement and interaction essential for active learning. Despite online team-based learning (TBL) offering potential solutions through structured cooperative activities, its efficacy in virtual simulation experiment courses remains scantily researched. This study investigates the effectiveness of online TBL for teaching virtual patient experiments in a basic medical laboratory course and contrasts it with traditional offline teaching in terms of student performance and perceptions.

**Methods:**

A comparative analysis involved 179 Year 3 medical students using online TBL, face-to-face TBL (FTF-TBL), and the flipped classroom (FC) approach. The learning outcomes were assessed based on experiment reports, IRAT scores, TRAT scores, and final exam performance. Students’ perceptions of both online and in-class TBL methodologies were also surveyed.

**Results:**

Both online and in-class TBL groups demonstrated comparable academic outcomes and surpassed the FC group in academic performance. Students displayed a marked preference for the TBL format (whether online or in-class), valuing its enhancement of learning interest and practical knowledge application. Nevertheless, refinements in discussion efficiency, platform convenience, and student-instructor interaction were indicated as potential areas of improvement in the online setting.

**Conclusions:**

Online TBL, along with its in-class counterpart, showed superior academic performance and a more positive learning experience compared to the FC group. These findings underscore the potential of online TBL in adapting to modern pedagogical challenges and enriching medical education through virtual simulation experiments.

## Introduction

Technological advancements are rapidly transforming medical and health education, providing rich learning environments, immersive learning experiences, and opportunities for self-paced or supplementary learning [1, 2]. The COVID-19 pandemic accelerated this transformation, triggering an urgent global shift from traditional face-to-face (FTF) teaching to virtual remote learning in academic institutions. This emergency transition has brought about unique challenges in course delivery, including adapting traditional teaching methods for effective online instruction, struggles to keep students engaged and motivated, and preserving sufficient social interaction among learners and instructors [3, 4].

To address these challenges, the fully online flipped classroom (FC) model has been touted as a promising strategy. Prior research has demonstrated that the conventional FC model—where students learn basic concepts online before class and engage in face-to-face activities during class—typically outperforms traditional teaching methods in enhancing student performance and engagement [5–7]. However, during the pandemic, several studies reported that students in online flipped classrooms did not achieve superior learning outcomes compared to those in traditional teaching settings, and some students expressed lower satisfaction with instructor motivation and feedback [8–12]. These findings underscore the need for enhanced interaction and communication in online learning environments.

Team-based learning (TBL) is a pedagogical approach centered on peer collaboration, a feature that inherently enhances interaction among students. By employing small-group instruction, TBL promotes active engagement in knowledge application and critical problem-solving skills [13, 14]. One notable advantage of this approach is the immediate feedback, initiated by peers and reinforced by the instructor, which has been shown to specifically enhance certain learning outcomes. These outcomes include improved problem-solving abilities, heightened critical thinking skills, and a deeper understanding of course material. Furthermore, TBL has been observed to bolster students’ self-efficacy and promote self-directed learning [15, 16]. These cognitive and behavioral changes, complemented by TBL’s collaborative nature, result in increased student engagement, satisfaction, and effective teamwork. These specific gains underscore TBL’s effectiveness, particularly in combination with flipped classroom (FC) methods [17–20].

Recently, online TBL has also been successfully implemented [21, 22], but research on its effectiveness remains limited. Babenko et al. found that in-person and online TBL classes resulted in comparable learning of student clinical reasoning during a family medicine clerkship [23]. A study from different scientific disciplines discovered that TBL online synchronized instruction achieved the same level of student approval as traditional FTF-based TBL instruction [24]. Interestingly, in another study of an undergraduate immunology course, although they confirmed an equal performance between the in-class and online TBL formats, they revealed a student preference for the in-class approach [25]. Shoair et al. reported similar favorable perceptions of FTF-TBL compared to online TBL in pharmacy students [26]. These diverse findings call for more research on the effectiveness of online TBL, as well as the critical factors that may influence student perceptions, such as course characteristics, contexts, and the dynamic nature of virtual learning environments.

While traditional theoretical courses are typically conducted offline, virtual simulation laboratory courses not only address the challenges of hands-on activities in a virtual setting but also offer students a comprehensive online experience from content to format [27, 28]. Among various simulation tools, the use of virtual patient (VP)—screen-based interactive scenarios designed to simulate real-world challenges—has gained prominence [29, 30]. This trend has been particularly evident in China, where the incorporation of virtual simulation experiment courses into medical education has seen steady growth since the Ministry of Education established the National Virtual Simulation Experimental Teaching Center in 2013 [31, 32]. These VP-based experiments stand out for their ability to integrate real-time human physiological parameters into virtual systems, thereby not only enhancing students’ application of knowledge and clinical reasoning abilities but also making it possible to conduct complex human physiology experiments virtually [33, 34].

Without the constraints of physical space, all aspects of VP experiments can be conducted remotely, making online experimental teaching feasible. However, current research has yet to compare the learning effectiveness of VP experiments conducted either in-class or online. In addition, although online TBL works well in theoretical courses, its suitability and effectiveness for VP experiments have not yet been explored. Given the increasing reliance on online education in the medical field and the unique pedagogical requirements of laboratory courses, understanding the comparative effectiveness of these instructional approaches is of immediate importance. Therefore, the aim of this study is to assess the effectiveness of online TBL as a new pedagogical tool for teaching VP experiments to medical undergraduates, and to compare student performance and perceptions in online TBL versus traditional offline teaching.

## Methods

### Design and participants

At Zhejiang University School of Medicine, third-year medical students were enrolled in a mandatory basic medical laboratory course, encompassing modules on inquiry-based animal experiments, human physiological experiments, and VP experiments. The course was delivered using an FC approach, with class sizes ranging from 28 to 32 students. Within these modules, the VP experiment module adopted FC or TBL strategies. However, due to the impact of the COVID-19 pandemic between 2021 and 2022, some classes transitioned to an online TBL delivery mode. The selection of online TBL, FTF-TBL, and FC groups for comparison was strategically made to evaluate the adaptability and effectiveness of these pedagogical approaches under the unique constraints of remote learning during the pandemic. This comparison aims to provide a comprehensive analysis of how different instructional methods impact student learning outcomes in a rapidly changing educational landscape. For the purpose of this study, 179 medical students, determined through convenience sampling, were grouped into the online TBL (*n* = 61), FTF-TBL (*n* = 56), or FC group (*n* = 62) in the VP experiment module. After the class, students are required to complete experiment reports. A final examination assessing both factual recall and application of knowledge from all modules is administered at the end of this course. All procedures in this study received approval from the Ethics Committee of Zhejiang University School of Medicine (IRB 2023-002), and informed consent was obtained from all participants.

### Structure of FTF-TBL and online TBL

**Fig. 1:**
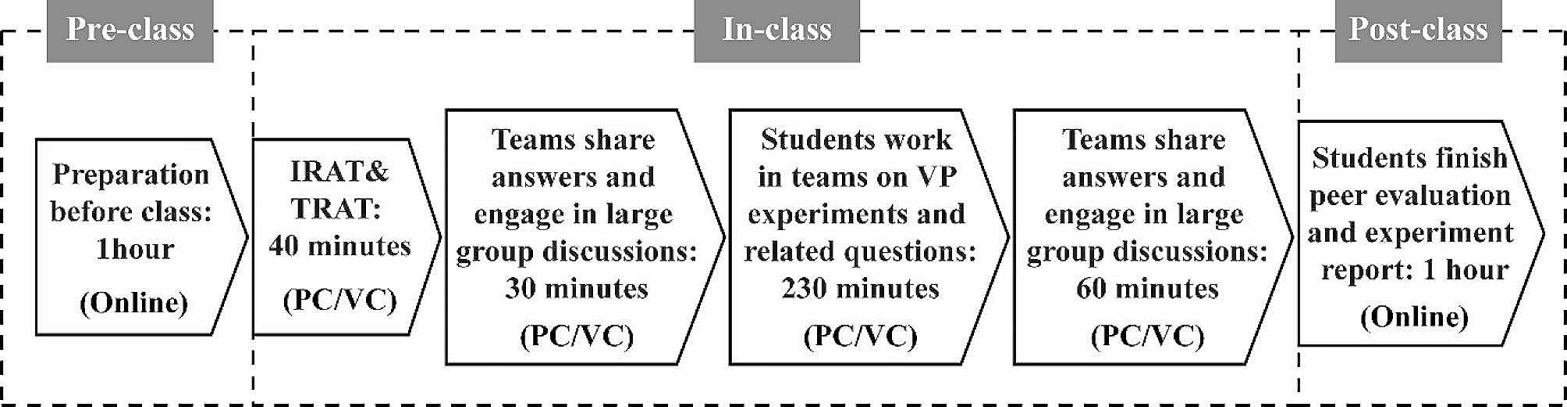
An overview of the administration and sequence of activities of FTF-TBL and online TBL. PC, Physical Classroom; VC, Virtual Classroom

The VP module encompasses two respiratory system-related experiments: “Regulation of Respiratory Movement” and “Diagnosis and Treatment of Chronic Obstructive Pulmonary Disease”. Student teams, consisting of five to six members, were carefully curated by the researchers to ensure a diverse composition within each team. The students were also asked to choose a team leader. Consequently, a class was formed by combining five such teams. These teams and the class configuration remained unchanged throughout the module. Instruction for all FTF-TBL and online TBL classes was provided by two content experts experienced in TBL pedagogy, with one having undergone training from the Team-Based Learning Collaborative (TBLC). An illustrative representation of the two methods in practice is shown in Fig. 1.

#### FTF-TBL

Before class, students accessed online materials, including pre-recorded lectures, slides, and readings to prepare for in-class activities. In class, students first took a 15-minute Individual Readiness Assurance Test (IRAT) with 15 multiple-choice questions. This was followed by a team version, the Team Readiness Assurance Test (TRAT), using scratch cards for 25 min. Teams then shared answers, leading into a 30-minute instructor-led discussion for clarity and addressing disagreements. Next, students engaged in 290 min of application activities. They collaborated on virtual patient experiments that include immediate feedback on experimental operations, and tackled five questions related to the comprehensive analysis of experiment results for 230 min, followed by a 60-minute large group discussion based on the 4 S principles (significant problem, same problem, specific choice, and simultaneous reporting). Post-class, students submitted experiment reports and evaluated peers on team contributions, though these peer scores didn’t impact final grades.

#### Online TBL

We adhered to online TBL guidelines proposed by Malik et al. [35]. The same online resources provided to the FTF-TBL group were available to students pre-class. Dedicated software facilitated the simultaneous online execution of both the readiness assurance process and application exercises. All these tools had been previously employed for online classes, so students were familiar with them. The students were further oriented about the use of the software, particularly for the use of breakout rooms. The online TBL sequence was as follows: First, the IRAT was conducted remotely via METESP software (Tencent, Shenzhen, China). Then the students moved to breakout rooms in DingTalk platform (Alibaba Group, Hangzhou, China) for the TRAT, collaborating within their TBL teams. After discussing among team members and reaching a team consensus, team leaders submitted answers through METESP. Then all the students went to the main DingTalk room to have a large group discussion led by the instructor. For the application exercises, teams returned to breakout rooms, working on virtual patient experiments and addressing related questions. Upon completion, students went back to the main DingTalk room, presenting their responses simultaneously using DingTalk’s screen-sharing feature. The instructor facilitated the large group discussion, prompting students to elaborate on their selections. The experiment reports and peer evaluation requirements after class are the same as for FTF-TBL.

### Structure of FC

Instructors consistent with both FTF-TBL and online TBL also led the traditional FC teaching. Although students in the FC group accessed the same pre-class resources as their TBL counterparts, they were additionally tasked with preparing presentations addressing experiment-related questions, encompassing basic knowledge, procedures, and anticipated experimental results. During class, students began with a pre-test which has the same questions as the IRAT from the TBL format. Subsequently, the instructor randomly selected a representative from each experimental operation group to make presentations based on pre-prepared queries. Peer discussions and queries were encouraged, with instructors stepping in for clarification and summarizing key takeaways at the end of the presentation. Students then individually undertook the virtual patient experiments and engaged in a subsequent question-and-answer session. After class, students are required to complete the experiment reports.

### Data collection and analysis

Scores from IRAT, TRAT, experiment reports, and final exams across all groups were aggregated and analyzed using SPSS 26.0. Upon completing the VP experiment module, all students filled out a feedback questionnaire featuring five items on a five-point Likert scale (1 = strongly disagree; 5 = strongly agree), designed to evaluate their engagement, satisfaction, and perception of the learning experience in different teaching methods. The selection of these specific items aimed to capture students’ subjective experiences and perceptions, providing insights into the impact of TBL and FC approaches on student learning. Additional insights were gathered from the online TBL group about their virtual learning experiences. Data distribution normality was evaluated using the Kolmogorov-Smirnov test. The Kruskal-Wallis test was used to analyze the differences between groups for non-normally distributed data. Cronbach’s alpha assessed the tool’s reliability. A *P*-value below 0.05 denoted statistical significance. Thematic analysis of student questionnaire responses was conducted through an inductive approach. Initial themes were identified by the first author and refined in collaboration with the second author to ensure consistent interpretation. A coding framework, emergent from the data, was then applied across the dataset by the research team. This process enabled the authentic capture of student perspectives, with representative quotes selected for their relevance and clarity [36].

## Results

### Student performance

**Fig. 2:**
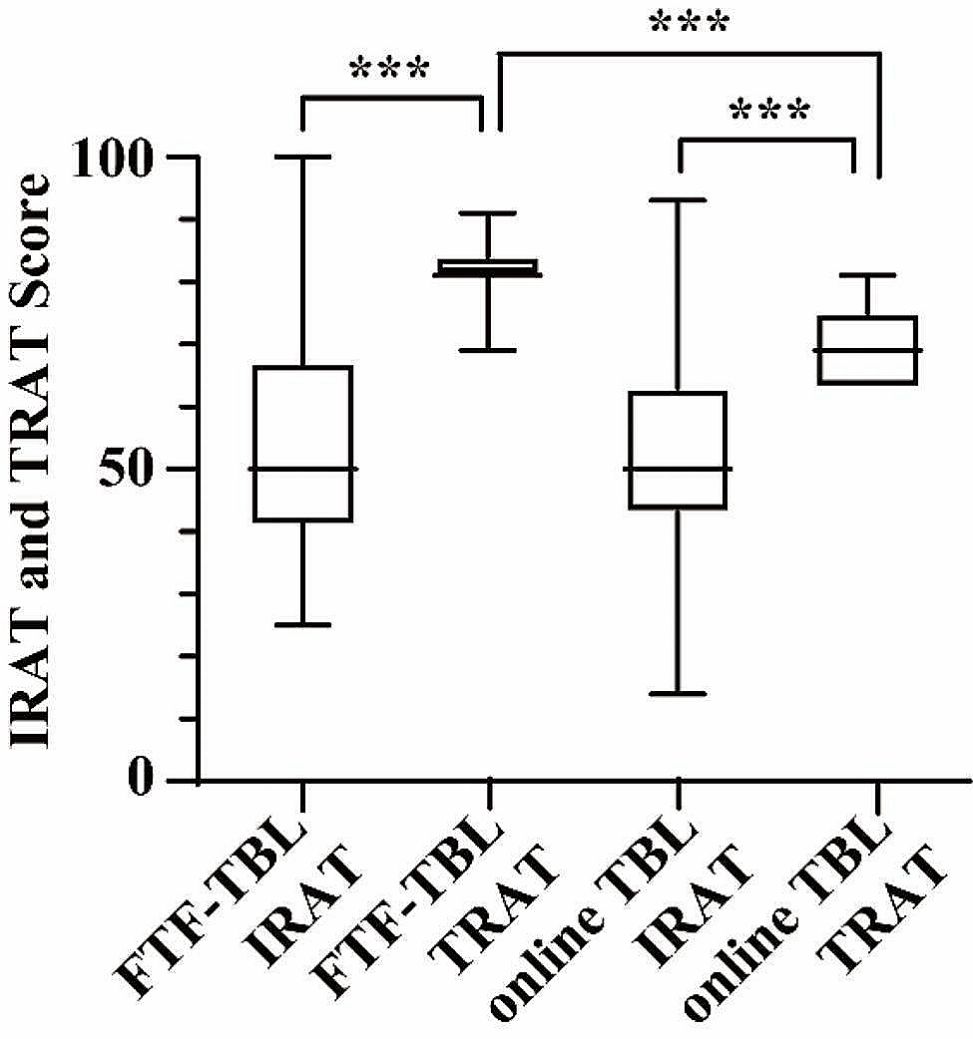
Comparison of the IRAT and TRAT scores between the FTF-TBL and online TBL groups. ***P < 0.001, compared with the IRAT scores in the same group or the TRAT scores in the different group

**Fig. 3:**
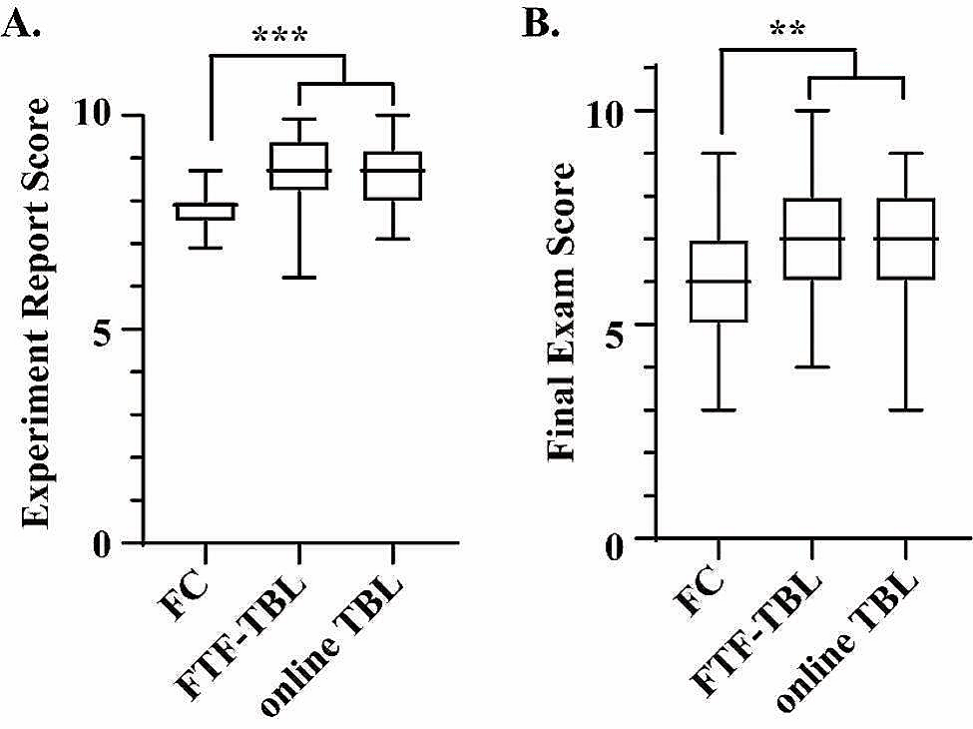
Comparison of the experiment report **(A)** and final exam scores **(B)** of all participants in three groups. ***P < 0.001, **P < 0.01, compared with the FC group

Figure 2 displays a comparison of the IRAT and TRAT scores between the FTF-TBL and online TBL groups. While the median TRAT scores outperformed the IRAT scores in both groups, the online TBL group’s median TRAT score was notably lower than that of the FTF-TBL group, despite similar IRAT scores. Upon assessing the academic performance of all participants in three groups, the FTF-TBL and online TBL groups demonstrated comparable scores in experiment reports and final exams, yet both outperformed the FC group, as illustrated in Fig. 3.

### Student satisfaction

Of the 179 students across the three groups, 166 (representing a 92.7% response rate) completed the questionnaire on their experiences with FTF-TBL, online TBL, or FC. The results of each group were non-normally distributed according to the Kolmogorov-Smirnov test. The Cronbach’s *α* coefficient of the questionnaire is 0.84. As outlined in Table 1, students exposed to both FTF-TBL and online TBL methods reported these approaches as superior to FC in terms of stimulating their learning interest, enhancing learning efficiency, and improving their practical knowledge application. In general, students found FTF-TBL and online TBL more challenging than FC but were more satisfied with the TBL approaches. Comparing the two TBL groups, online TBL students felt their method was slightly less efficient and more challenging than what FTF-TBL students reported.

**Table 1:** Comparison of students’ perceptions of FC, FTF-TBL and online TBL

Survey item	FC, *n* = 62 Median (IQR)	FTF-TBL, *n* = 56 Median (IQR)	online TBL, *n* = 61 Median (IQR)	*P* value^a^		
				FTF-TBL vs. FC	online TBL vs. FC	online TBL vs. FTF-TBL
The course has improved my interest in learning	3.5 (3–4)	5 (4–5)	5 (4–5)	**< 0.001**	**< 0.001**	0.999
The course has improved my learning efficiency	4 (3–4)	5 (5–5)	5 (4–5)	**< 0.001**	**< 0.001**	**0.041**
The course has improved my ability to apply knowledge in practice	4 (3–4)	5 (5–5)	5 (4–5)	**< 0.001**	**< 0.001**	0.504
I find the teaching method used in the course to be challenging	2.5 (2–3)	4 (3–5)	5 (4–5)	**< 0.001**	**< 0.001**	**0.048**
Overall, I am satisfied with the learning experience	3 (3–4)	5 (4–5)	5 (4–5)	**< 0.001**	**< 0.001**	0.506

FC = flipped classroom; FTF = face-to-face; TBL = team-based learning

a = pairwise comparisons using the Dunn test after a significant Kruskal-Wallis result (*P* < 0.05)

Likert Scale; 1 = strongly disagree, 2 = disagree, 3 = neither agree nor disagree, 4 = agree, 5 = strongly agree

To better understand student views on virtual learning, we asked those in the online TBL group to compare their online experience with their prior offline TBL sessions from different modules within the same course. Of the 61 students, 51 responded: 49.0% (25/51) favored FTF-TBL, 23.5% (12/51) found online TBL superior, and 27.5% (14/51) regarded both formats as equally effective. For the open question “What areas do you believe need improvement in online TBL?”, four themes were generated from the codes: “efficiency of discussions”, “convenience of the online platform”, “student engagement”, and “interaction with the instructor”.

#### Efficiency of discussions

Almost a third of the respondents (33.3%, 17/51) felt that the cooperation efficiency of team discussion in online TBL could be enhanced.

*It is not timely for team members to enter the breakout room, and the communication efficiency is not as good as face-to-face.*

*Compared with offline, team members need more time to fully understand each other’s ideas, affecting the efficiency of cooperation.*

*We sometimes waited for each other to speak first, and time quickly passed. This led to a decrease in the efficiency of the discussion.*

### Convenience of the online platform

Of the respondents, 17.6% (9/51) indicated that the software and platform usability could be improved.

*Video conferencing is not easy to switch.*

*The window label of the video conference is not clear enough to identify the group members.*

*When making a mind map, only one student can operate it, and other team members cannot cooperate to edit it.*

### Student engagement and interaction with the instructor

Five students (9.8%, 5/51) reported insufficient engagement in group discussions. Some described that *a few students did not speak*, and some students found it *awkward to speak in an online environment*. Two students (3.9%, 2/51) expressed a desire for increased communication with the instructor. They found that they *could not consult the instructor in time when they encountered problems in the experiment*, and they also voiced a desire for the instructor to *step in and provide guidance when team discussions were insufficient*.

## Discussion

Since the push for reform in medical education, accelerated by the pandemic crisis, online teaching and learning across all areas of medical and healthcare education has significantly increased [37, 38]. This shift has forced institutions to confront new challenges, such as recreating the in-class interactions and dynamics which are considered essential for engaging students in active learning instruction. With the help of the highly structured form of small group learning, online TBL provides students with opportunities to apply conceptual knowledge through active cooperative learning activities [21–23, 25]. However, there is limited research on the effectiveness of online implementation of such instructional strategy, particularly in the context of virtual simulation experiment courses. Therefore, in this study, we aimed to investigate the educational impact of online TBL in a Year 3 basic medical laboratory course focusing on virtual patient-based experiments. Our results show that students in the online TBL group performed as well as those in the in-class TBL group, and better than the FC group. Overall, students expressed greater satisfaction with the TBL method regardless of the environment (online or face-to-face), but they also suggested room for improvement in the online version.

Our previous research discovered that a blended learning model combining FC with TBL led to better academic outcomes and higher student satisfaction than flipped teaching alone in medical laboratory teaching [17]. Interestingly, in the present study, we further found that online TBL retained the effectiveness of its in-class counterpart in fully virtual experiments. These results align with earlier studies [23, 25], which suggest that students can successfully perform team-based activities in both online and in-class settings, with no significant difference in learning outcomes. An intriguing observation in our study was that while the IRAT scores were comparable between the online TBL and FTF-TBL groups, the TRAT scores were significantly lower in the online group. This hints at potential differences in readiness between the two settings. However, these disparities seemed to be resolved following the group application activities, including VP experiments and group discussions, as we observed no differences in the quality of experiment reports or final exam scores between the two groups.

Some studies have highlighted the role of technological proficiency for effective participation in online learning [26, 39]. While our students were comfortable with video conferencing platforms, largely due to the widespread use of online lectures, they were less familiar with online group discussion tools. This gap in familiarity could affect the quality and depth of discussions during the TRAT. Nevertheless, in the subsequent VP experiments that were focused on task-oriented learning and case study problem-solving, teachers noted more active and engaged discussions among students in the breakout rooms. This heightened level of engagement, therefore, could improve both the quality of student discussions and their sense of social interdependence [40].

In student surveys, both online and in-class TBL were perceived as more effective and satisfying than FC in stimulating learning interest, enhancing efficiency, and improving practical knowledge application. Several factors may contribute to the high levels of satisfaction observed with our online TBL approach. Firstly, it has been suggested that robust faculty development and support could offer benefits like adapting existing programs for online delivery, curating resources, and fostering faculty collaboration [41, 42]. To ensure effective practice, we consulted with experienced TBLC instructors and followed established guidelines for online flipped learning, such as developing the orientation session pre-class, discussing each question one by one in the class, and using online tools to implement the 4 S principles [35, 43]. Secondly, most participants, including teachers and students, were relatively proficient in using different online platforms. This technological familiarity likely contributed to the smooth transition to online learning, though there’s room for further investigation to measure its specific impact on student satisfaction and learning outcomes. Thirdly, our task-oriented virtual patient experiments allowed students to tackle real-world problems and apply their theoretical knowledge in practical settings. This not only fostered positive interdependence by encouraging task-sharing among students but also helped to mitigate feelings of isolation that can sometimes accompany online learning [44].

While the online TBL approach was generally well-received by students, it is important to address the areas identified for improvement to optimize the overall learning experience. In the survey, some students pointed out specific aspects that could be enhanced, including the “efficiency of discussions”, “convenience of the online platform”, and “student engagement and interaction with the instructor”. It has been reported that students often need an adjustment period to engage effectively in online group discussions [45]. Given that our intervention spanned only a brief period of two weeks, students might not have had adequate time to acclimate to the online setting, thereby affecting the efficiency of discussions. Furthermore, effective time management and self-discipline are crucial for meaningful participation in virtual TBL [26]. Students should set objectives, manage tasks wisely, and allocate time efficiently to meet deadlines and contribute effectively to team progress. Incorporating relevant content into the pre-class orientation session could enhance the efficiency of online discussions and learning. To enhance the convenience of the online platform, introducing features like collaboratively editable documents could be beneficial. Additionally, a comprehensive platform that encompasses all facets of online TBL—including video conferencing, RAT, and group discussions—may be worth developing in the future [22]. As for the issue of student engagement and interaction with the instructor, several strategies could be employed to enhance the digital learning environment. Strengthening the existing peer evaluation process can further minimize the “free rider” phenomenon among students, thereby fostering a heightened sense of responsibility and engagement. Instructors could also focus on creating a safe environment where students feel comfortable answering questions without fear of ridicule or blame. Additionally, instructors might increase their visits to the breakout discussion rooms to offer real-time assistance and feedback, thereby promoting more effective learning. Furthermore, future research could benefit from a deeper exploration of the facilitator’s role in online TBL settings. Effective facilitation, particularly in navigating online platforms and maintaining student participation, is crucial for TBL’s success. The facilitator’s ability to manage online discussions and provide timely feedback can significantly influence student engagement and learning outcomes. Recognizing and optimizing the facilitator’s role in virtual environments will be an important aspect of future research, especially given the rapid shift to online education.

The current study offers valuable insights into optimizing online TBL for facilitating laboratory courses. This is particularly useful for settings where there are a large number of students but limited faculty resources, or where logistical challenges exist. This educational approach not only equips students with essential skills for collaborating and learning in a digital environment but also prepares them for a healthcare sector increasingly reliant on remote and online services. The comparative analysis of online and in-class TBL in this study contributes to the novel understanding of how different teaching methodologies impact student learning, particularly in virtual simulation environments. Our findings provide a fresh perspective on the adaptability and effectiveness of TBL strategies, enriching the dialogue on educational methods in the digital age.

### Limitations

This study presents several limitations warranting consideration. The focus was solely on virtual patient experiments related to the respiratory system, which may introduce a specific bias related to the source of knowledge. Coupled with the absence of random group assignments, this narrows the generalizability of the findings. Additionally, the research’s short duration could have impacted the outcomes, especially in terms of long-term retention and knowledge applicability. It’s also important to note that the study was conducted during the pandemic, a time when students were already accustomed to online learning. This could have influenced their receptiveness to the online TBL methodology. These aspects suggest that the observed differences might not solely be attributable to the instructional methods used but could also be influenced by these contextual factors. Future research addressing these constraints is necessary to provide a more comprehensive understanding of the relative effectiveness and implications of different instructional approaches in virtual simulation courses.

## Conclusion

Our comparative analysis revealed that both online and in-class TBL strategies outperformed the FC method in a virtual simulation experiments setting. Students demonstrated comparable academic performance in both TBL environments, with no significant difference in learning outcomes. The online TBL approach achieved similar scores in experiment reports and final exams as its face-to-face counterpart. Students exhibited a preference for the TBL format—whether online or in-class—over FC teaching for its ability to enhance learning interest and the practical application of knowledge. Nonetheless, they also pinpointed areas for refinement in the online version, including the need for improvements in discussion efficiency, platform convenience, and student-instructor interaction. Overall, our study underscores the adaptability and effectiveness of the online TBL, especially its capacity to nurture collaboration in virtual environments, priming students for challenges in the evolving realm of digital healthcare and education.

## Data Availability

Datasets underpinning this study’s conclusions are provided in the article. While individual student data isn’t publicly accessible to protect student privacy, they can be obtained from the corresponding author upon a reasonable request.

## Abbreviations

FC: Flipped classroom
FTF: Face-to-face
IRAT: Individual readiness assurance test
TBL: Team-based learning
TRAT: Team readiness assurance test
VP: Virtual patient
